# Comparison between subjective and objective methods to assess functional capacity during clinical treatment in patients with intermittent claudication

**DOI:** 10.1590/S1679-45082013000400016

**Published:** 2013

**Authors:** Gabriel Grizzo Cucato, Antônio Eduardo Zerati, Marcel da Rocha Chehuen, Raphael Mendes Ritti-Dias, Glauco Saez, Luciana Ragazzo, Pedro Puech-Leão, Nelson Wolosker

**Affiliations:** 1 Hospital Israelita Albert Einstein, São Paulo, SP, Brazil; 2 Hospital das Clínicas, Faculdade de Medicina, Universidade de São Paulo, São Paulo, SP, Brazil; 3 Physical Education an Sport School, Universidade de São Paulo, São Paulo, SP, Brazil; 4 Universidade de Pernambuco, Recife, PE, Brazil

**Keywords:** Peripheral arterial disease/therapy, Exercise test, Walking

## Abstract

**Objective:**

To analyze if there is any relation between functional capacity assessed by subjective and objective methods regarding the current state and after clinical treatment in patients with intermittent claudication.

**Methods:**

A total of 500 patients with intermittent claudication were enrolled. All patients underwent clinical examination and a functional evaluation by subjective (clinical visit) and objective method (treadmill test). Additionally, 50 patients were selected to evaluate the effect of clinical treatment by subjective and objective methods in relation to functional capacity.

**Results:**

Out of 500 patients, only 60 (12.0%) had similar results in both methods. The remaining, that is 440 patients (88.0%) had subject values in disagreement with stress test findings. Regarding the clinical effect of the treatment on the functional outcomes, results were similar in both methods (χ^2^=1.7; p=0.427).

**Conclusion:**

Although the subjective method overestimates the functional capacity when compared to the objective method, no significant differences were observed between both methods when analyzing the effect of clinical treatment. Thus, the subjective method provides similar information as compared with objective method, in monitoring the clinical treatment of patients with intermittent claudication.

## RESUMO

**Objetivo:**

Verificar se há relação entre a medida da capacidade funcional avaliada subjetiva e objetivamente em relação ao estado atual e após tratamento clínico em pacientes com claudicação intermitente.

**Métodos:**

Foram recrutados 500 pacientes com claudicação intermitente. Todos realizaram exame clínico e foram submetidos a uma avaliação funcional por meio do método subjetivo (consulta clínica) e objetivo (teste de esteira). Adicionalmente, 50 pacientes foram selecionados para se avaliar o efeito do tratamento clínico, pelos métodos subjetivo e objetivo, em relação à capacidade funcional.

**Resultados:**

Dos 500 pacientes selecionados, somente 60 (12,0%) apresentaram valores referidos e observados similares. Os demais pacientes, ou seja, 440 (88,0%) apresentaram valores relatados discordantes em relação àqueles obtidos no teste de esforço. Com relação ao efeito do tratamento clínico em relação à capacidade funcional, os resultados foram similares entre ambos os métodos (χ^2^=1,7; p=0,427).

**Conclusão:**

Apesar de o método subjetivo superestimar os valores de capacidade funcional, quando comparados ao método objetivo, não foram observadas diferenças significantes entre ambos os métodos, quando se analisou o efeito do tratamento clínico. Assim, o método subjetivo fornece informações similares em comparação à medida objetiva no acompanhamento do tratamento clínico de pacientes com claudicação intermitente.

## INTRODUCTION

Peripheral artery disease (PAD) includes a series of structural dysfunctions leading to reduced lumen of trunk vessels in the lower limbs, mostly because of atherosclerosis^(1)^, which affects approximately 10.5% of the Brazilian population aged over 18 years^(2)^. One important feature of PAD is the decreased peripheral artery blood flow, which frequently leads to symptoms of pain, cramps, burning or muscular fatigue when walking^(3–5)^. This set of symptoms is known as intermittent claudication (IC) and it affects 25 to 33% of individuals with PAD^(4,5)^.

The presence of IC symptoms results in reduced physical activities^(6)^, thus interfering in functional capacity^(7)^, health and quality of life^(8)^, and ultimately death due to cardiovascular disease^(9,10)^. On the other hand, with the purpose of promoting benefits to these patients, physical exercise, associated with drug treatment, is the initial medical treatment recommended for them, considering their significant results in improving functional capacity^(11)^, quality of life^(12)^ and associated comorbidities^(13,14)^, with surgical^(15)^ or endovascular^(16)^ treatment being performed only when clinical treatment does not work effectively.

Because of marked functional limitation imposed by the IC symptoms, measurements of claudication distance and total walking distance (TWD) have been widely used as clinical indicators^(4,5)^. Until a few years ago, evaluation of these measurements was carried out only subjectively, *e.g.*, distances walked depended only on the distance reported by the patients, which left doubts in regard to the method reliability, in addition to the measuring the maximum distance walked, especially for scientific evaluation of data. Subsequently in an attempt to obtain more precise and objective data, standardized tests on a treadmill started being used, and nowadays they are considered the gold standard for evaluating the functional capacity and following up of clinical parameters of patients with IC^(17)^.

However, treadmill tests cannot always be offered at healthcare units because of their operational cost and required time to perform; therefore, values of functional capacity obtained through TWD reported by the patients remain widely used in the clinical practice, with objective tests being relegated only to groups that scientifically investigate this illness. However, it is still not clear if the functional capacity figures obtained by this method are different than those quantified by the objective method. For this reason, it is necessary to investigate if both methods provide similar information in regard to the clinical progression of patients submitted to treatment, thus helping physicians choose the type of management for this type of patient.

## OBJECTIVE

To compare the functional capacity responses assessed by the subjective method (values reported by the patients) and quantified by the objective method (values quantified in a treadmill test); to analyze if both assessment methods present similar values in regard to functional capacity in patients submitted to clinical treatment.

## METHODS

In the period between December 2008 and December 2012, patients with PAD and symptoms of IC were enrolled at the Department of Vascular Surgery, of *Hospital das Clínicas da Faculdade de Medicina da Universidade de São Paulo* (USP). This was a prospective, non-randomized and uncontrolled study. All patients enrolled were submitted to the same experimental protocol in accordance with the standards of the Ethics Committee on Human Research of *Hospital das Clínicas da Faculdade de Medicina da USP,* under number 121,001.

The inclusion criteria were patients should be able to walk on a treadmill or have a medical history of experience on this ergometer. Patients excluded from the study were those with severe coronary artery disease, arterial hypertension or decompensated *diabetes mellitus,* and those submitted to revascularization surgery less than one year ago. All patients enrolled underwent a clinical examination, medical history taking, ankle-brachial index (ABI), and objective and subjective evaluations of a TWD.

### Diagnosis of peripheral artery disease

Diagnosis of PAD was made based on the following criteria: (1) ABI at rest ≤0.90 in one or two limbs; (2) palpation of peripheral artery pulses (femoral, popliteal, posterior tibial and pedal); (3) Fontaine II Classification, with the presence of CI symptoms, such as pain, cramps, burning and tingling in lower limbs during the walking exercise test^(4)^.

### Assessment of functional capacity

For subjective assessment of functional capacity, during a routine medical visit the patients were asked by the investigator about their estimate of maximum walking capacity through the following question: “What is the maximum distance that you think you can walk until the symptom of pain prevents you from continuing the exercise?”.

For objective assessment of the functional capacity, patients were submitted to a walking exercise test on a treadmill by using a specific protocol for this population^(17)^. The test was carried out at a constant velocity of 3.2km/h and increments of 2% in slope every 2 minutes, until the patient could no longer walk due to the symptoms of pain. The test was interrupted when the patients could no longer stand the pain and, at that moment, TWD was recorded (in meters).

### Clinical treatment and subjective and objective assessments of functional capacity

Of the 500 patients initially enrolled, a sub-sample of 50 patients was selected with the purpose of analyzing the effect of clinical treatment by means of subjective and objective methods in relation to functional capacity. These patients were instructed to carry out clinical treatment in accordance with the international and national guidelines for the disease^(4,5,18)^, which recommend the control of associated comorbidities (hypertension, *diabetes mellitus*, hypercholesterolemia and smoking habit) and to do physical activities every day, preferably walking for one hour, aiming at reaching the total walking distance in the presence of pain (TWD). After three months of treatment, these patients returned to hospital and they were reassessed by both methods (subjective and objective).

### Statistical analysis

For data analysis, responses were considered similar between the objective and subjective methods when the values did not exceed 30 meters. For analysis of the association between subjective and objective tests of the functional capacity and to quantify the changes with clinical treatment, a χ^2^ with p<0.05.was used.

## RESULTS

Of the enrolled patients, 500 met the initially proposed criteria and they were included in the study. Of those, 60% (300/500) were male, with mean age of 62 years (32 to 88 years), 75.5% had a history of smoking habit, and a past history of several comorbidities associated with PAD, such as hypertension (63.0%), *diabetes mellitus* (27.5%), angina (19.5%) and acute myocardial infarction (17.5%).

In regard to the answers of functional capacity between both methods, of the 500 patients included in the study, only 60 (12.0%) presented similar values in regard to TWD between the subjective and objective methods. The other patients, *e.g.*, 440 (88.0%) presented discrepant reported values in relation to those obtained in the walking test on a treadmill, and in 147 patients (29.4%) the reported value was higher than the one observed and, in 293 patients (58.6%) the reported value was lower than the one observed. These values are shown in table 1.

**Table 1:** Comparison of the values obtained in outpatient visits in patients with intermittent claudication (n=500)

Comparison of methods	n (%)
Subjective = objective	60 (12.0)
Subjective > objective	147 (29.4)
Subjective < objective	293 (58.6)
Total	500 (100)

Regarding the effect of clinical treatment on functional capacity, in subjective assessment, out of the 50 patients selected, 31 (62%) reported improvement, 12 (24%) worsening and 7 (14%) reported stability of functional capacity after three months of clinical treatment with 34 patients (68%) presenting improvement, 7 (14%) patients presenting worsening and 9 (18%) patients presenting stability when assessed by the objective method. Additionally, the effects of clinical treatment in functional capacity were similar in the objective and subjective methods (χ^2^=1.7; p=0.427) (Table 2).

**Table 2:** Comparison of the progress of functional capacity in patients with intermittent claudication after 3 months of clinical treatment

Progress assessment	Subjective n (%)	Objective n (%)
Improvement	31 (62)	34 (68)
Worsening	12 (24)	7 (14)
Stability	7 (14)	9 (18)
Total	50 (100)	50 (100)

## DISCUSSION

The main findings in this study were: (1) values of functional capacity obtained through the subjective method showed differences in relation to those obtained by the objective measurement. (2) However, subjective and objective methods showed similar results in regard to the effect of clinical treatment in relation to the functional capacity in patients with IC. The results of the current study indicated that the functional capacity obtained through the subjective method, was different from the measurement obtained with the objective method for more than 80% of the patients. These results are not surprising since previous studies reported a weak relation^(19,20)^ between values assessed by the subjective and objective methods of functional capacity. Therefore, some hypotheses can be raised to explain the possible discrepancies between the different methods for assessing functional capacity. It is possible that the specific feature of this disease, *e.g.*, the symptoms of IC, present in daily physical activities of these patients, may drastically decrease the quality of life and health perception, while when asked about his/her TWD, these patients can report underestimated values of walking to the physician^(19)^. Additionally, since most of the patient's daily activities are carried out on flat surface, these features are different from those of the walking exercise test used, which was progressive and carried out on a treadmill at a fixed velocity of 3.2km with increments of 2% in slope per minute. Lastly, the physical exertion carried out on a treadmill can be quite different from walking during daily physical activities and it can be an additional explanation for the differences observed in relation to the TWD between the two methods.

On the other hand, although the absolute values of TWD have been different between the subjective and objective methods, the results related to the follow-up of patients clinical course showed good agreement between them, suggesting that the use of the subjective method can be an alternative way for the follow-up of clinical progress in patients with IC. The main possible explanation for these results would be that the patient, while undergoing clinical treatment (walking), has a great possibility of increasing his/her TWD and this may reflect on improved perception of pain and/or health leading to better quality of life; these factors could be reported to the physician during the clinical consultation. On the other hand, when the patient does not undergo clinical treatment, it is possible that his/her TWD decreases, resulting in worsening of quality of life and self-perception of health, which would affect his/her response during the clinical consultation^(21)^.

The results of this study may have important clinical application, since several health centers do not have specific structure for setting up an ergometric assessment room or skilled professionals. In addition, a treadmill test requires time for being performed, which may not be feasible at some vascular surgery centers due to the high number of patients seen every day. Therefore, functional capacity assessed by the subjective method can be an interesting alternative to monitor the patient's clinical status in view of several types of treatment used in clinical practice. Additionally, qualitative assessment (based on clinical progress) can be considered enough for the physician to follow up a patient with claudication, especially in cases of reported clinical improvement, without the need of using methods of higher operational cost for the treatment (treadmill).

This study has some limitations. The use of only one question with the subjective method may have been insufficient to assess the patient's current status of functional capacity^(19)^. In fact, estimation of the functional capacity of walking can be influenced by several external factors such as walking velocity, place where the patient lives and unilateral or bilateral disease – these factors are important and they are used in this study. In addition, comparison between the subjective and objective methods applied to the walking exercise test may present important bias since the exercise performed on a treadmill (constant velocity and progressive increase in slope) can be quite different from the patient's daily walking activities. Lastly, not using questionnaires that assess quality of life did not allow assessment of the determinants that could explain the similar results between the methods observed after the clinical treatment.

## CONCLUSION

The subjective method underestimated the values of functional capacity when compared with the objective method. However, after clinical treatment, both methods presented similar values in regard to functional capacity. Therefore, the subjective method (functional capacity reported by the patient) can be an alternative tool for the follow-up and progress of clinical parameters of patients with IC submitted to clinical treatment.
